# Correction of Alar Retraction by Articulated Alar Rim Graft Combined with V-Y Advancement

**DOI:** 10.1007/s00266-025-05506-3

**Published:** 2025-12-16

**Authors:** Linglong Zhan, Xuming Wang

**Affiliations:** Department of Plastic and Reconstructive Surgery, Chongqing Dangdai Plastic Surgery Hospital, No. 3, 2 nd Branch, North Jianxin Road, Jiangbei District, Chongqing, 400020 China

**Keywords:** Alar retraction, Alar rim graft, V-Y advancement, Revision rhinoplasty

## Abstract

**Background:**

The alar-columellar relationship was crucial for aesthetic evaluation of the lower nasal area, with alar retraction being a common and challenging deformity in Asian rhinoplasty.

**Objectives:**

This study aims to evaluate the effectiveness of articulated alar rim graft (AARG) combined with V-Y advancement in correcting alar retraction.

**Methods:**

A retrospective analysis was conducted on 25 patients (48 sides of alae) who underwent revision rhinoplasty using AARG combined with V-Y advancement between September 2021 and June 2022. Postoperative photograph was used to follow-up with the patients. The distance from the alar rim to the nostril axis was measured preoperatively and postoperatively to assess the correction effectiveness.

**Results:**

The mean follow-up period was 8 months. The preoperative distance from the alar rim to the nostril axis averaged 3.4 mm, reduced to 1.5 mm postoperatively. The average reduction was 1.9 mm. Most patients exhibited natural soft tissue contours postoperatively, with no significant graft-related complications.

**Conclusions:**

The combination of AARG and V-Y advancement is effective in correcting alar retraction, maintaining aesthetic nasal contours, and preventing long-term alar deformities.

**Level of Evidence IV:**

This journal requires that authors assign a level of evidence to each article. For a full description of these Evidence-Based Medicine ratings, please refer to the Table of Contents or the online Instructions to Authors  www.springer.com/00266.

## Introduction

The alar-columellar relationship plays an important role in the aesthetic evaluation of the lower nasal area. Gunter et al. first categorized the alar-columellar relationship into six types, of which type II retracted ala is particularly common in Asian rhinoplasty and is still considered one of the challenging nasal deformity [1]. The alar retraction (AR) is diagnosed when the distance between the alar rim and the long axis of the nostril is greater than 2 mm in the lateral view. Due to the complexity of alar retraction, there is no consensus on its classification. Hirohi et al. classified two types of alar retraction morphologically: Type I is a completely displacement of the alar rim to the inferior border of the lower lateral cartilage, and the nasal cavity can be seen from the frontal view. Type II includes those cases where part of the alar rim is supero-laterally displaced and appears to be notched [2]. Kim et al. classified the alar retraction based on alar notching into type 1 (the medial type), type 2 (the central type), and type 3 (the lateral type) [3]. Depending on the degree of alar retraction, the appropriate method should be chosen. Mild retraction of 1 to 3 mm may be corrected by placement of an alar contour graft. Moderate to severe retraction (more than 3 mm) requires more aggressive techniques for correction such as the caudal repositioning of the lateral crus, lateral crural strut grafts (LCSG), intercartilaginous grafts, alar batten grafts, alar spreader grafts, articulated alar rim grafts (AARG) and so on [3–8]. For nasal retraction caused by a shortage of vestibular lining, commonly used methods include auricular composite grafts and V-Y advancement [9]. Alar retraction caused by more than one factor, including but not limited to excessive resection of the cephalic border of the lateral crus, scar contracture and vestibular lining defects, is common in clinical practice and is difficult to correct by a single approach.

We note that the previous study by davis et al. on AARG is impressive in that AARG not only corrects alar retraction, restores the aesthetic appearance of the alar ridge, and prevents deformity of the alar rim in the postoperative long-term follow-up, but also is beneficial for nasal airway patency [10]. In solving the problem of inadequate nasal vestibular lining, the auricular composite grafts are effective but there are various problems such as auricular deformity, additional scarring and necrosis of the skin cover of the cartilage graft. The V-Y advancement is superior in terms of concealment and acceptance in Asian patient by comparison. Therefore, articulated alar rim graft combined with V-Y advancement is used in the correction of alar retraction in Asians. Based on our experience, this method is effective while maintaining an aesthetic nasal contour and, more importantly, avoiding alar deformity in the distant postoperative period.

## Patients and Methods

We retrospectively analyzed 25 patients (including 48 sides of alae) who underwent revision rhinoplasty from September 2021 to June 2022. Including 3 males and 22 females were treated with the method of AARG combined with V-Y advancement. The age of the patients ranged from 18 to 45 years, with a mean age of 31 years. All patients had undergone one or more rhinoplasties. Preoperative physical examination revealed varying degrees of iatrogenic alar rim retraction, with no presence of airway obstruction. Exclusion Criteria: (1) History of nasal trauma. (2) Congenital nasal deformities (e.g., cleft lip nasal deformity). (3) History of non-surgical treatments for nasal deformities (e.g., filler injections). (4) Severe systemic diseases contraindicating surgery. (5) Pregnant or lactating. (6) Patients with psychiatric conditions unable to cooperate with follow-up. Based on the classification for alar retraction proposed by Kim et al., 19 patients (76%) had type 1, 4 patients (16%) had type 2, and 2 patients (8%) had type 3. The surgeries were performed by the same senior surgeon.

### Operative Technique

Appropriate amounts of costal cartilage were excised as needed with general endotracheal anesthesia. Through the external approach, an inverted V-shaped incision was used for the nasal columella, and a marginal incision was made along the caudal border of the lower lateral cartilage. Unusually, a “V” shaped mucosal flap was designed starting from the apex of the nostril. The incision was extended cephalically to the intercartilagious line. The tip of the “V” was angled toward the most posterior portion of the nostril and was in the vertical line of the most concave part of the alar rim. A mucous flap was developed on the subperichondrial level and was extended caudally (Fig. 1). Degloving of the lateral crus was performed from the dome, followed by complete degloving of the middle vault including release of the vertical scroll ligament. After lysis of cephalic adhesions and unfurling of the contractured internal lining, all patients underwent both columellar strut and extended spread graft (ESG) placement as part of the treatment protocol. Then the lateral crural steal (LSC) was used to raise and relocate the domal apex. This program was named as lateral crural tensioning (LCT). The AARG was fabricated from the bone cortex of rib cartilage, which was thin and narrow (Fig. 2). AARGs typically measured 20 to 25 mm in length, 5 mm in width, and 1 mm in thickness. The medial border was positioned flush with the tip defining point (TDP), which diverges from the nasal midline at a 45 degrees angle, to ensure the graft being fixed on TPD without unwanted increases in tip projection. The medial and cephalic graft edges were beveled for seamless camouflage. The remaining graft was tapered laterally, with the caudal edge always parallel to the alar rims. A modest graft convexity was also used to provide proper support to alar. Two fixations of the AARG to the lateral crus were performed by 5-0 PDS. The third suture is placed on the TPD (Fig. 3). Then a precise intracutaneous pocket was dissected along the lateral aspect of the alar rim, beginning where the graft diverges from the lateral crus and extending into the central alar lobule. The lateral aspect of the AARG was inserted into the pocket without fold.

**Fig. 1:**
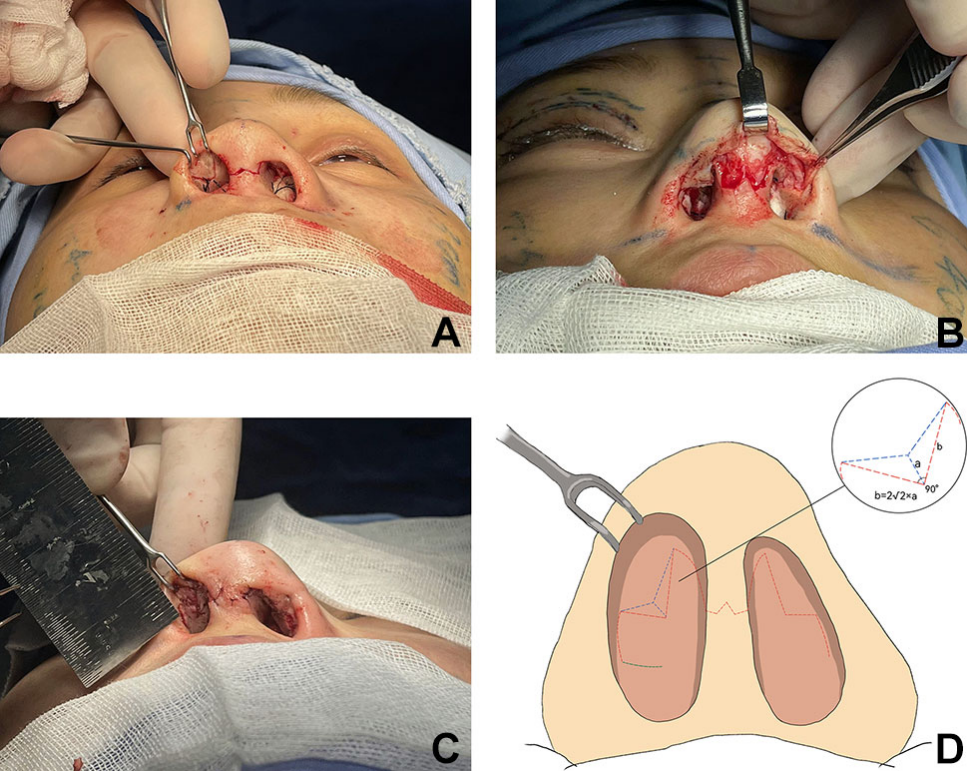
Revision of alar retraction using internal V-Y advancement. **A** Intraoperative photo of the V-shaped incision of the nasal mucosa. **B** The V flap is unfolded like a book page after dissection. **C** The Y advancement is sewn in position. **D** Diagrammatic illustration of operative incision (dashed red line), extended incision (dashed green line), and the Y advancement sewn in position (dashed blue line)

**Fig. 2:**
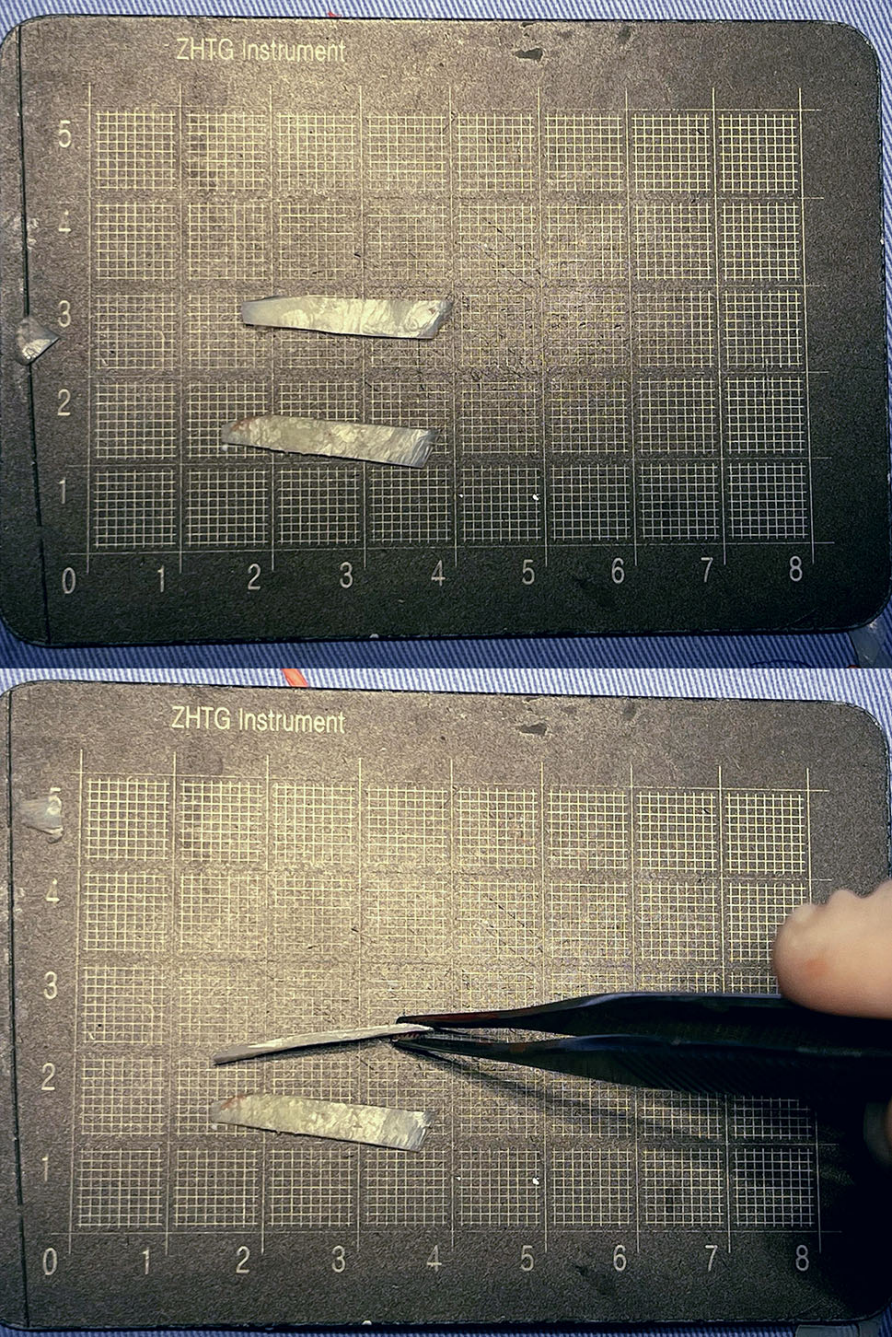
Typical contour of articulated alar rim grafts (AARGs). The medial edge is carved at a 45-degree angle to ensure smooth articulation with the tip defining point (TDP)

**Fig. 3:**
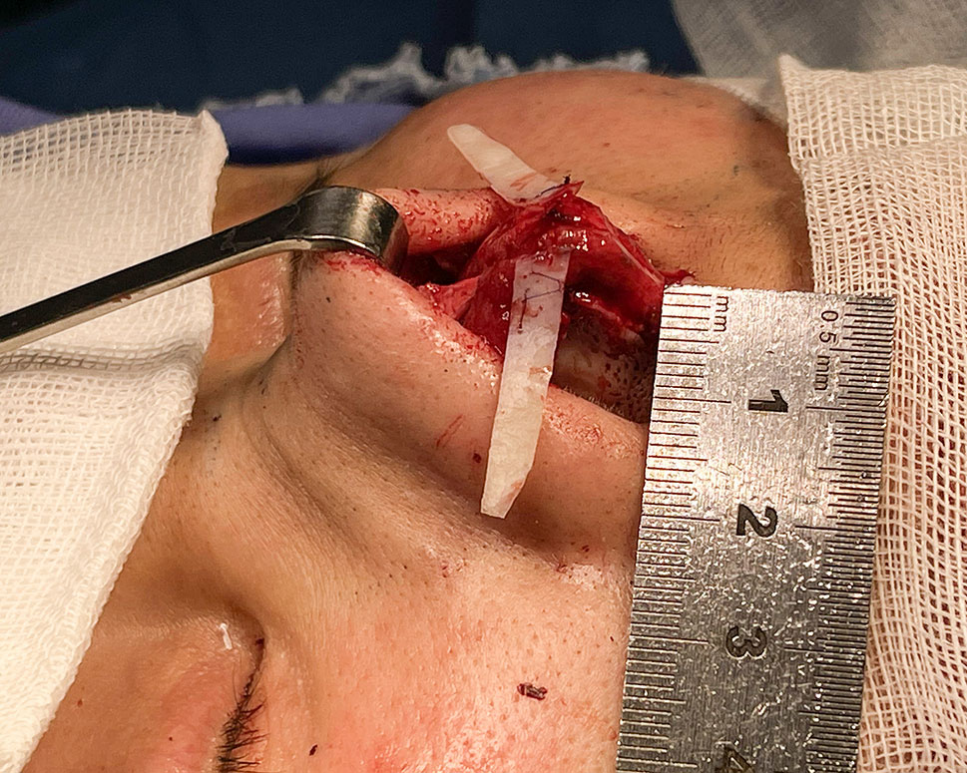
Articulated alar rim graft (AARG) fixation.Note fixation to both the lateral crus and the tip defining point (TDP)

After closing the incision in the nasal columella, the flap that was incised V and dissected was folded further caudally, and a Y advancement was accomplished (Fig. 1). The left incision was closed meticulously to prevent graft exposure. The alar complex was sandwiched in between two flexible circular sheets. These sheets were supported in position using a through-and-through 3-0 nylon “U” stitch, which is removed in 5 days. It should be noted that do not tie the suture tightly. The nostrils were supported with silicone tubing wrapped in petroleum jelly gauze for more than 7 days.

### Photographic Analysis

All patients were photographed preoperatively and postoperatively, by the same equipment under standardized protocols. The patient’s head was positioned parallel to the Frankfort horizontal plane to maintain a natural head position. The distance between the alar rim and the long axis of the nostril was in the lateral view and was measured before and after surgery to assess the improvement (Fig. 6).

### Statistical Analysis

The statistical analysis was performed using SPSS. We used the paired t-test, with *P*-values less than 0.05 considered to indicate statistical significance, to evaluate the differences between the preoperative and postoperative ratios.

## Results

All patients showed natural soft tissue contours of the nostrils and alae after the operation (Figs. 4 and 5). The mean follow-up interval was 8 months, with a range of 4 to 17 months. The preoperative distance from the alar rim to the long axis of the nostril was ranged from 2.7 to 4.5 mm in the lateral view, with a mean value of 3.4 mm, whereas the postoperative distance was ranged from 1.2 to 1.9 mm in the lateral view, with a mean value of 1.5 mm (3.4 mm vs. 1.5 mm, *P* < 0.001). The distance between the alar rim and the long axis of the nostril was reduced on average by 1.9 mm (range, 1.1 to 2.6 mm) in the lateral view. One patient developed moderate bilateral alar retraction at the 4-month follow-up. Given the patient’s history of nasal contracture due to previous rhinoplasty infection, we believed this complication was related to that prior event, although no obvious signs of infection were observed during follow-up. Two patients had partial but significant improvement with some mild rim deformities. There were no visible grafts, graft displacements, or graft infections observed (Fig. 6).

**Fig. 4:**
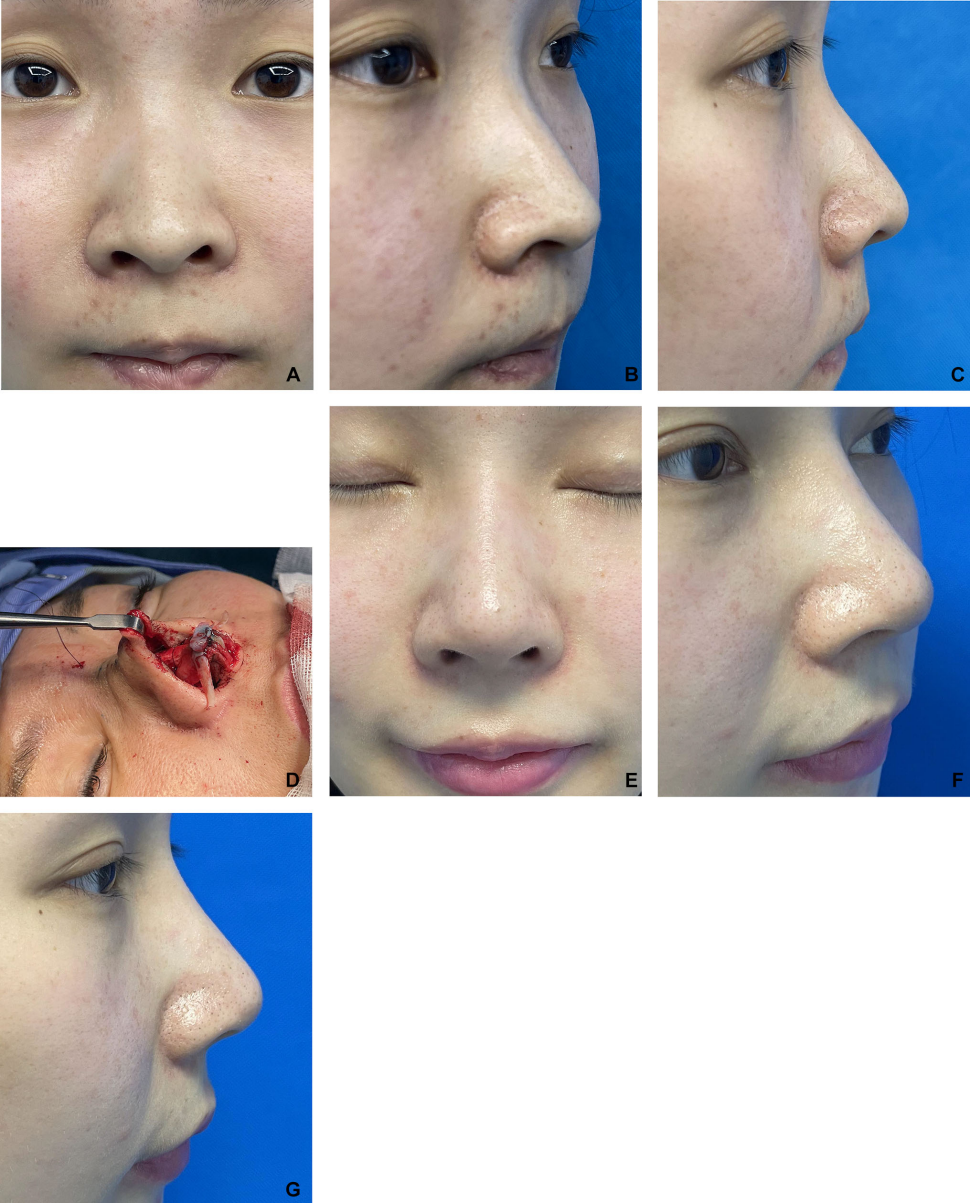
A 28-year-old female. **A**, **B**, **C** Preoperative photos. **D** Intraoperative photo showing the articulated alar rim graft (AARG) sewn to the septal extension graft at the tip defining point (TDP). The other grafts include columellar strut, extended spread graft(ESG), and onlay tip graft. **E**, **F**, **G** 6 month after operation

**Fig. 5:**
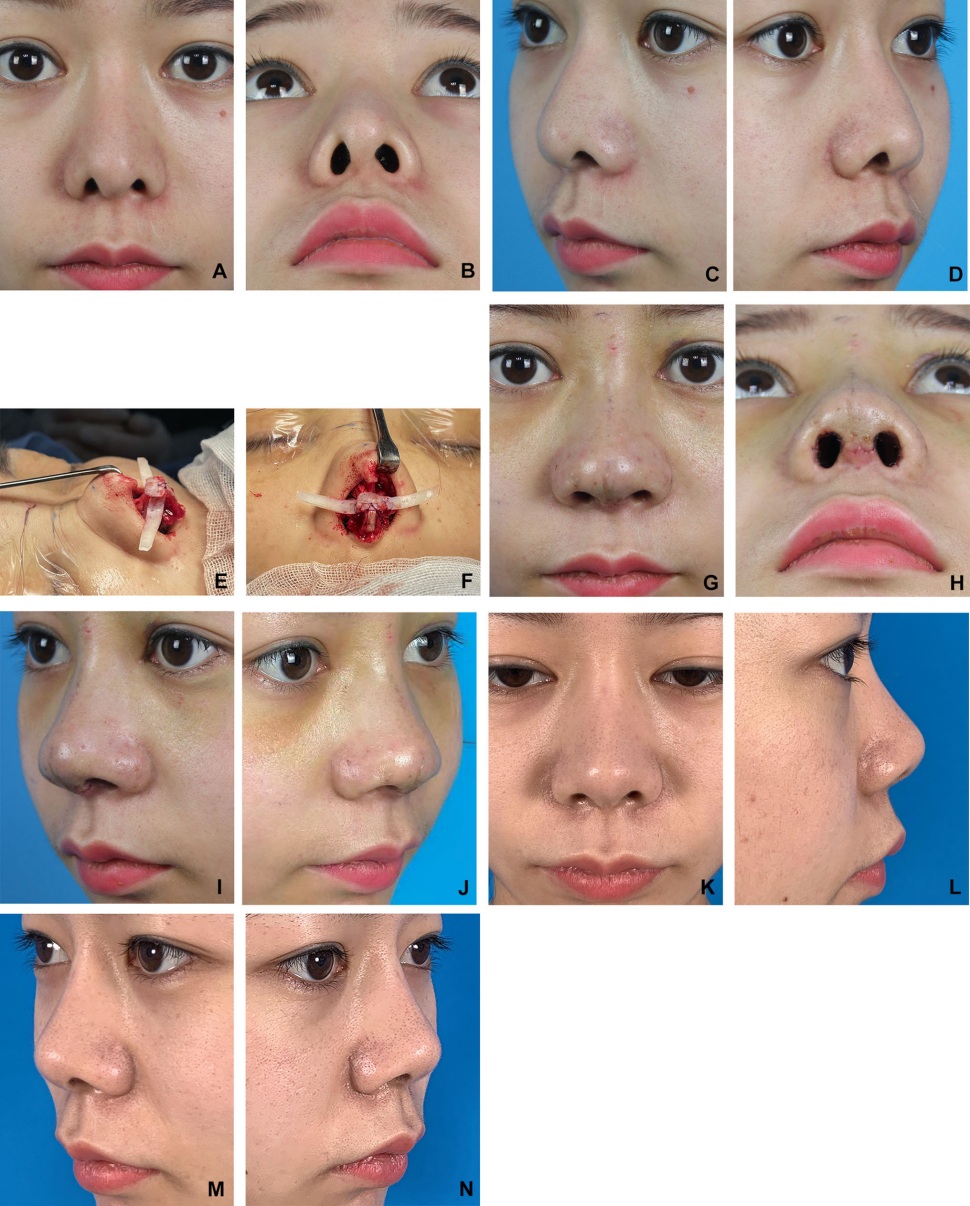
A 31-year-old female. **A** to **D** Preoperative photos. **E**, **F** Intraoperative photo showing the articulated alar rim graft (AARG) sewn to the septal extension graft at the tip defining point (TDP). The other grafts include columellar strut, extended spread graft(ESG), shield graft, and onlay tip graft. **G** to **J** 10 days after operation. **K** to **N** 8 month after operation

**Fig. 6:**
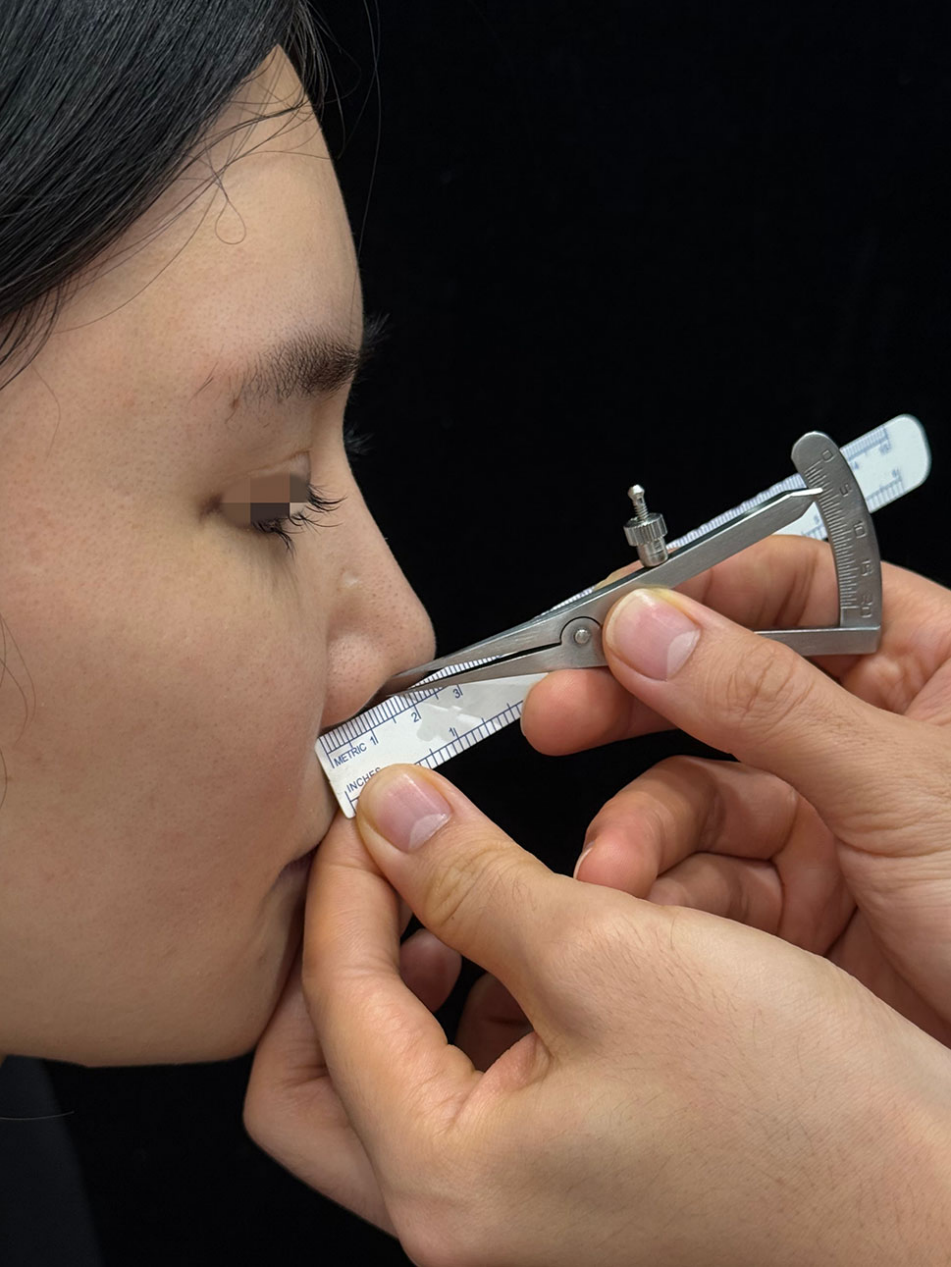
Measurement method for alar rim retraction. The distance between the alar rim and the long axis of the nostril in the lateral view was measured using a divider

## Discussion

The alar retraction is difficult to repair due to the lack of support of the alar rim and the defective and inelastic nasal vestibular lining. Conventional methods have limitations to some extent: (1) Alar contour graft is too weak to cope with moderate or severe alar retraction, with the risk of retraction in the long term. (2) Lateral crural strut graft and alar batten graft, both designed to strengthen the lateral crural, differ in that the former are located on the deep side of the lateral crural while the latter are located on the surface. They can’t provide any support for the soft tissue of alar rim so that they can’t correct the aesthetic defects of the alar rim. Even worse, there is a risk of internal nasal valve impingement from the LCSG. (3) Intercartilaginous graft placement requires the remnant of the lateral LCSG to be strong enough. This puts higher demands on doctors’ skills in terms of the selection of the grafts, their sculpting, placement, and the dissection of the operative area. (4) Auricular composite graft will cause an additional incision which is unacceptable by some patients. It depends on the local blood supply of the recipient area for survival. The graft which is overlarge or placed inappropriately will roll the nasal lining out of the vestibule and create the odd appearance of a double alar rim. The AARG combined with V-Y advancement solves these problems to some extent. The AARG which is longer and wider than the alar contour grafts provides greater support by connected with the TDP and lower lateral cartilage. The strength of this structure can avoid cephalic displacement of the graft. Because AARG is kind of nonanatomical graft placed 2 to 3 mm above the nostril rim, it has a more direct shaping power [11]. AARG can be placed with simple sutures without the need for complex mechanical equilibrium. Adjacent tissue used to reconstruct nasal lining by V-Y advancement brings better aesthetics. Comparing with the auricular composite graft, V-Y advancement is easier to master and apply.

The length of AARGs depends on both the nasal size and the classification of alar retraction. It must fully bridge the tip and alar lobules while also extending beyond the highest point of the alar notching. If it is too long, it may enter the pyriform aperture resulting in the foreign body sensation and alar stiffness. In this study, since the majority of our patients presented with iatrogenic alar retraction—primarily type 1 and type 2—the AARGs did not require excessive length to adequately cover the retracted area. For severe alar retraction, the width of AARGs can be appropriately increased to enhance the force countering the retraction. Regarding the selection of surgical indications, due to the limitations in the patient selection for this study, the surgical method may be more suitable for AR type 1 and AR type 2. Nevertheless, efficacy was evident in the only 2 type 3 cases, an outcome that may be attributed to the correction of soft tissue deficiency via the V-Y advancement.

We prefer to use rib cartilage cortex as the material, which is more adequate, flat and flexible compared to septal and auricular cartilage. Before sculpting, the rib cartilage was soaked in saline for more than 15 minutes. After its natural bending deformation, we can select the part with appropriate length and curvature. The cartilage cortex on one side of the rib is sliced up and bisected along the long axis to ensure that the curvature of these two grafts is basically the same. The high flexibility of the rib cartilage cortex allows the graft to be made thinner overall, but the cephalic graft edges should be still beveled for seamless camouflage.

Before discussing the technical details further, we would like to emphasize the importance of LCT. LCT is the combination of LCS and septal extension graft (SEG). Since Asian rhinoplasty tends to increase the height of the nasal tip and dorsum, we place strut and ESG (a type of SEG) in all patients and complete the LCS by attaching the lower lateral cartilages to the SEG using suture techniques. Revision the sidewall tension is the greatest contribution of the LCT, which provides the following benefits: minimizing unsightly sidewall pinching, enlarging internal nasal valve dimensions to improve ventilatory disorder and opposing upward displacement of LCC from scar contracture to prevent alar retraction. LCT is the basis for the effectiveness and stability of the AARG [11]. We usually use three-point fixation to ensure that the AARG does not displace. Two points of fixation are at the medial-most end of the graft and at the point of divergence from the lateral cartilage. Now, the AARG is positioned at 90 degrees to the sagittal midline. The third point connects the medial end of the graft to the nasal tip like an articulation. Tip grafts like shield grafts and onlay tip grafts are always necessary in Asian rhinoplasty to highlight the TDP and placed before AARG. After their placement, unlike previous authors, the primary reason why the medial side of the AARG is sculpted at 45 degrees is to avoid adding tip volume rather than to modify tip appearance.

The preparation of the intracutaneous pocket is important equally. We believe that the following three points should be emphasized: (1) The pocket size should be adapted to the graft. A small pocket will lead to lateral curling of the graft. (2) Try to dissect the pocket as close as possible to the alar rim. This will sufficiently release the bound of the edge to descend. (3) Expose the nasal vestibule with a double hook retractor. Press the skin with one finger and feel the position of scissor tip at the same time to avoid perforating skin. When AARG is inserted into the pocket, alar shape has been changed even though it is made as thin as possible. Ballin et al. had named the “alar ridge” to the flat and narrow ridgeline located immediately cephalad to the alar margin which is connect the TDP with alar lobules [10]. We also observed this subtle effect of the AARG on the alar rim. AARG with an increase in the thickness of the medial portion and a greater convexity is more beneficial to the patients who have both lobular pinching and alar retraction.

An internal V to Y advancement had been successful in the senior author’s (BG) practice for the first time in 2001. The need for this technique should be evaluated during the preoperative design of the incision line. The angle of V flap should be around 90 degrees. The side length of V flap depends on the alar retraction severity. According to rough mathematical calculation (Fig. 1D), the side length should be at least twice the distance that we expect to reduce between the alar rim and the nostril long axis to account for flap extensibility. In practice, we usually design a side length of 7-8 mm for patient with 3-mm retraction of alar. Guyuron et al. concluded that retractions of as much as 5 mm can be corrected with this technique. This is generally consistent with our experience because the side length of V flap cannot be extended infinitely [9]. As the internal V-Y incision is repaired, the lateral nasal wall cephalic to the alar rim becomes tighter. In patients with severe shortage of nasal vestibular lining, V-Y advancement may result in the lateral incision of V flap not being able to be sutured for excessive tension. Therefore, we extent the lateral incision of V flap downward and cephalad until the incision parallel to the alar rim can be closed by a local rotational flap (Fig. 1). The small defect in the extended incision can self-heal in 3–5 days without suture. Since this defect area is far from the cavity where the dorsal nasal prosthesis is placed, there is no increased risk of prosthesis-related infection.

In conclusion, articulated alar rim graft combined with V-Y advancement can utilize their full advantages to correct alar retraction effectively. The two techniques do not interfere with each other during the surgery and do not add operative difficulty. With this procedure, reconstructing alar appearance and obtaining patient satisfaction is possible. Certainly, this study has certain limitations, primarily due to the relatively small sample size and generally short follow-up period. Its single-center retrospective design may introduce selection bias.
